# Hybrid Seeds in History and Historiography

**DOI:** 10.1086/721075

**Published:** 2022-09

**Authors:** Helen Anne Curry

**Affiliations:** Department of History and Philosophy of Science, University of Cambridge, Cambridge CB2 3RH, United Kingdom

## Abstract

Accounts of twentieth-century agricultural industrialization in the United States and beyond often center the production and distribution of commercial F1 hybrid seed as a pivotal development. The commercialization of hybrid corn seed in the 1930s was initially heralded as a science-driven advance in agricultural productivity. However, since the 1970s “hybrid seed” has been linked to many perceived perils attendant on industrialized agriculture, from the undermining of farmers’ independence to the diminishment of crop genetic diversity to the consolidation of corporate control over the global food system. First grouped with the semidwarf varieties of the Green Revolution to emblematize capital- and chemical-intensive agriculture, hybrids are today often lumped together with genetically modified (GM) varieties for much the same reason. This essay revisits the scholarship that helped produce this understanding of hybrid seed. It explores how and why the singular history of hybrid corn inflected understandings of crop breeding and seed production in general, contributing to lasting confusion about the promises and pitfalls of distinct approaches to crop development and the nature of hybrid seed.

Accounts of twentieth-century agricultural industrialization in the United States and beyond often point to the production and distribution of hybrid seeds as a pivotal development.^1^ There’s good reason for this emphasis. The successful commercialization of F1 hybrid corn seeds in the United States contributed to a profound transformation in the role played by public scientists in crop development and paved the way towards a transnational seed industry.^2^ And these are not the only crop hybrids of historical renown. The semidwarf wheat and rice varieties that underpinned the late-twentieth century agricultural transformations known as the Green Revolution have also been described at times as hybrids.^3^

Building on these associations, critics since the 1970s have blamed “hybrid seeds” for social and environmental problems arising from industrialized agriculture. Their accounts describe how hybrid seeds undermined farmers’ independence and livelihoods, diminished crop genetic diversity, and encouraged consolidation of corporate control over the global food system.^4^ This straightforward depiction of an agricultural evil unsurprisingly has its own critics, and not just among agroindustry allies. In a 1996 analysis of vague agricultural terminology muddying decisionmaking in agricultural development, the anthropologist Robert Tripp observed that “no term is more misused … than ‘hybrid.’”^5^ Tripp was concerned that this term had come to be associated almost exclusively with F1 hybrids (a concept I explain further below) and, despite often being categorized as such, the semidwarf wheat and rice varieties of the Green Revolution were *not* F1 hybrids.

Tripp, drawing on the influential analysis of the sociologist Jack Kloppenburg, located the roots of this confusion in a linguistic shift arising from the commercialization of hybrid corn seeds. In the 1930s and 40s, a general meaning of hybridization as “the cross breeding or sexual combination of two varieties” had narrowed to indicate the crossing of two inbred lines to produce F1 seeds as in the case of hybrid corn.^6^ In fact, the shifting meanings of “hybrid” among plant experts has a longer history, with an even older definition referring primarily to crosses between species.^7^ Nonetheless, the introduction of hybrid corn seed production marked a pivotal moment in the history of “hybrid” as a descriptor for plants. Now this label was linked not just to nature’s monsters or breeders’ artifices as in the Victorian era but also a new class of seeds—and, crucially, to the emerging systems of industrial research and production from which F1 hybrid seeds arose.

This association with industrial agriculture partly explains the elision observed by Tripp. In the case of Green Revolution-era semidwarf wheat and rice, their categorization as “hybrid” was less about the exact breeding techniques by which seeds were produced and more about the capital- and chemical-intensive style of agricultural production with which they were associated.

In this essay I reflect on the history and politics of “hybrid” as a description for seeds used in twentieth- and twenty-first-century agricultural production.^8^ I am inspired to do so by the contemporary association of hybrid seeds with genetically modified (GM) varieties—that is, varieties produced through recombinant DNA techniques or gene editing tools such as CRISPR/Cas9. These diverse products of professional plant breeding are often contrasted with open-pollinated, locally adapted varieties known variously as heirloom, traditional, heritage, or landrace crops. For example, the US heirloom vegetable conservation organization Seed Savers Exchange characterizes its offerings as “Heirloom, Untreated, Non-Hybrid, Non-GMO Seeds.”^9^

This elision, too, has frustrated experts. A quick internet search reveals dozens of pages that explain the differences between hybrid and genetically modified seeds.^10^ Many seem convinced that educating consumers about the biotechnologies of seed production will dispel misunderstandings that lead to the rejection of crops produced through “modern” breeding methods. What these analyses fail to see is that for decades hybrid has been less a description of a breeding technique and more a signifier of the political and technological modes of production with which a seed is associated—in this case, one associated (rightly or wrongly) with widespread use of chemical inputs, fossil fuel dependency, and control by transnational agribusinesses.

## Celebration and condemnation

Although, as I’ve mentioned, it’s possible to start a history of hybrid seeds and the controversies around them in the nineteenth century or even earlier, the debates that concern my story have twentieth-century origins. I begin with the event that Tripp understood as pivotal in determining the meaning of “hybrid” when used as a description for crop seeds or varieties: the development of hybrid corn.

The creation of a commercially viable system for producing hybrid corn seeds—a history I won’t recount in detail here as it’s been told often and, in some cases, very well indeed—was celebrated as a profound advance of modern scientific understanding and agricultural industry.^11^ Introduced in the United States in the 1920s, hybrid corn varieties overtook their open-pollinated predecessors at a rapid pace, constituting nearly 100 percent of cultivation in the Corn Belt by the end of the 1930s and nationwide by 1960.^12^ Corn yields per acre more than doubled in the same period, and about half of that increase is attributed to breeders crafting evermore productive hybrid varieties.^13^

Then, as now, hybrid corn varieties take advantage of the phenomenon of heterosis, also known as hybrid vigor. F1 hybrids are created by crossing genetically distinct parental lines that have been inbred over many generations and harvesting the “first filial” or F1 seeds that result from this cross. The F1 seeds in turn produce plants that may perform significantly better in the field than the lines from which either of their parents were derived. For geneticist James Crow, as for many others, figuring out how to harness heterosis to create higher-yielding crop varieties was “surely one of genetics’ greatest triumphs.”^14^

This triumph altered the relationship between US corn growers and their seeds. Vigorous F1 plants—that is, hybrid corn—generated higher yields but obliged farmers to return to a seed seller each year for a fresh supply. The genetic heterogeneity of the hybrids meant *their* offspring (the F2 generation) would be unlikely to show the same productivity. This characteristic made hybrid varieties appealing to seed companies, which would be rewarded for the extra labor needed to generate F1 seeds by the annual return of customers for a fresh supply. Success commercializing F1 hybrid corn seeds led professional breeders and seed companies to pursue the same strategy in other crops, at least where biology allowed.^15^

Biology did not always allow, however. Thanks to the high seed yields of corn (that is, the further number of seeds produced from planting a single seed) and the fact that it is an outcrossing plant whose cross-pollination is easily controlled, it was possible for companies to price F1 hybrid corn seeds such that they would profit *and* farmers would make up the premium they paid for the seeds through a larger harvest. By comparison, despite scientists’ best efforts, it was and remains challenging to develop commercially viable F1 hybrid seed systems for selfpollinating crops, including wheat and rice. In the 2010s, F1 hybrids represented only about 0.2 percent of global wheat acreage.^16^ Hybrid rice has been comparatively more successful. Chinese breeders developed the first commercially viable varieties in the 1970s; by the turn of the twentieth century, hybrids accounted for more than 50 percent of Chinese rice production and had begun to make inroads elsewhere.^17^

The Green Revolution wheat and rice varieties promulgated through international development programs in the 1960s and 70s were described as hybrids, typically by critics, almost from the moment of their introduction. But they were not F1 hybrids.^18^ What, then, did “hybrid” mean when applied to these varieties? One could reasonably argue that here the description captured the origin of these varieties in the hybridization of markedly distinct breeding lines. Drawing on this older and more general definition, “hybrid” here might refer, for example, to the recombination of tall Mexican wheat varieties with lines originating in Japan that carried a trait for short stature, the approach taken in creating the semidwarf Green Revolution wheat varieties.^19^

However, as Robert Tripp observed, there was more to characterizing Green Revolution seeds as hybrid than simple adoption of a wider definition. Thanks to its dominant association with F1 hybrids, this term “implie[d] serious restrictions on farmer seed saving.” When used as a description for the Green Revolution wheat and rice varieties, “hybrid” suggested that these seeds induced peasant farmers to abandon the practice of saving their own seeds—despite the fact that “[n]one of the rice or wheat varieties of the green revolution were [F1] hybrids… and evidence shows that farmers who plant M[odern] V[arieties] of these crops save the seed of a particular variety for many seasons.”^20^ In short, referring to Green Revolution seeds as hybrids was less a technical description and more a rhetorical move that positioned these ostensible products of international aid firmly in the realm of capitalist agroindustry.^21^

## Blighted hybrids

To understand why changes in seed saving practices would come in for scrutiny, it’s necessary to turn from questions of plant breeding method to those of intellectual property. I’m less interested here in recapitulating the debates over whether and how plant breeders should be allowed to secure intellectual property protections in their creations—the subject of another large, even exhaustive literature—and more interested in zeroing in the space that “hybrid seeds” occupied in these debates.^22^

In the 1970s, activists in the United States and Europe increasingly identified transnational capital as a chief source of problems in the global food system. For many, the dominance of big firms, and their concern only for the bottom line, explained global maldistribution of food, poor diets, and ecological degradation.^23^ A subset of these activists homed in on control over seed production as a key route through which the influence of transnational corporations in agriculture was deepening.^24^ Private industry had sought new means of exercising this control through intellectual property protections on crop varieties and were rewarded with the expansion of such protections in Europe (via the International Union for the Protection of New Varieties of Plants) and the United States (via the Plant Variety Protection Act) in the 1960s and early 70s.

Intellectual property in seeds thus became an important—and enduring—site of contestation for many activists. In his 1979 *Seeds of the Earth*, perhaps the most famous intervention into these debates, the Canadian activist Pat Mooney wove a complex narrative about the social and political changes that had led to ever-more-thorough dominance of industry in the supply of seeds to farmers and the social and environmental consequences of this new order. He dismissed the claimed benefits of the Green Revolution to peasant farmers and pointed instead to wealthy countries and, more specifically, transnational agribusinesses, as the real beneficiaries of agricultural industrialization in developing nations. Private seed companies had stepped in to produce and distribute new varieties. Those same companies had recently convinced many governments to expand the intellectual property protections to be given to plant varieties. Seed activists like Mooney linked the guarantees of expanded intellectual property systems to intensifying industry consolidation, in which large companies were buying out the small seed firms of an earlier era, to the loss of crop genetic diversity and ultimately to further erosion of poor farmers’ already limited autonomy through the control of seed production.^25^

For those who sought to understand and illustrate where greater private ownership of crop varieties would eventually lead, US hybrid corn, and F1 hybrids more generally, provided the obvious example. The production of hybrid seeds had appealed to private companies because it enabled them to exercise some control over the varieties they developed and sold even in the absence of formal intellectual property protections. Mooney claimed that this produced “the hybrid bias,” a tendency for companies to emphasize crops amenable to F1 hybrid seed production. As a result, “what is fundamentally a marketing consideration eventually influences the range of food varieties offered to people.”^26^ F1 hybrid technology had also made seed companies more powerful. A couple decades after the introduction of hybrids, a few corn seed companies had grown from modest family businesses into large corporations with significant economic clout.^27^ They also proved potent politically, arguing successfully against the release of new corn varieties by publicly funded programs as unfair competition, thereby diminishing public investment in and oversight of crop development. F1 hybrid seeds had paved the way for privatization.

In one of the most famous accounts of this progression, the sociologist Jack Kloppenburg outlined two transformative outcomes of the invention of hybrid corn. When seed production by private companies displaced on-farm production, the seed itself became a commodity. When these same firms shunted public programs to a supportive rather than competitive role, they established control over the form this commodity could take. The upshot, in Kloppenburg’s estimation, was a system that afforded less autonomy to farmers, diminished government research, transformed public investments in agriculture into subsidies for private companies, and reinforced the industrialized agriculture through the dissemination of varieties particularly suited to this style of farming.^28^

Critics like Mooney and Kloppenburg were also able to rely on the example of US hybrid corn to illustrate the hazards of an agricultural system possessing these features. In the 1960s, bountiful corn harvests were not only central to the agricultural economy but also a means by which politicians exercised “food power” over foreign leaders.^29^ This vaunted position intensified the real and perceived effects of a virulent fungal infection that swept US corn fields in 1970. The Southern Corn Leaf Blight dramatically reduced harvests that year, with economic losses estimated at more than one billion dollars (the equivalent of 6.25 billion today), and generated fears of feed shortages and escalating food prices.^30^ The blight was quickly linked to the widespread adoption of hybrid lines that possessed cytoplasmic genes for male sterility—a quality that made hybrids cheaper for seed companies to produce but held no other advantages.

That biological explanation of the blight quickly gave rise to more political ones. “The proliferation of brand name maize varieties had disguised the fundamental genetic uniformity of corporate seed corn development,” Mooney declared in 1979, a circumstance that had led farmers unwittingly to plant all the same lines at significant peril to their livelihoods. It substantiated his claim that “when these giant corporations direct their research dollars to hybrid development” the result was “increasing crop uniformity and genetic vulnerability.”^31^ And where some observers were quick to praise the fast response of the nation’s public agricultural research system to the blight, Kloppenburg countered it was this system’s “subordination to private enterprise” that had endangered the nation’s corn harvest in the first place.^32^

## Hybrid to GM

Kloppenburg’s analysis—famously, I would say, though perhaps that’s speaking as a historian of agriculture—deployed the history of hybrid corn not to speak to the perils of corporate control of food and agriculture in general but to deliver a specific message about biotechnology. Writing in the 1980s as the “new biotechnologies” of recombinant DNA and other molecular interventions approached commercial viability, he urged readers against “ceding to capital the exclusive power to determine how biotechnology is developed and deployed.” He predicted that biotechnologies used in breeding, bolstered by robust intellectual property protections, would “parallel the introduction of hybrid corn” in further commodifying the seed, giving industry power to determine the shape of the crop as well as its production system, and committing farmers to a relentless technological treadmill.^33^

Although I won’t go so far as to say that today’s confusion between F1 hybrids and the products of more recent biotechnologies traces to Kloppenburg, I do think that, like the confusion over the “hybrids” of the Green Revolution, they date to this period. They are a product of scholarship that deployed the history of F1 hybrid corn, and F1 hybrids more generally, as a cautionary lesson against the expansion of intellectual property rights in seeds in the 1970s and later. This scholarship—indebted to Marxist critiques of science under capitalism, entangled in international seed activism, and rendered unusually compelling by the 1970 corn blight epidemic—made hybrid corn seeds an incomparable example of the ills attendant on private industry’s control of seed production. It undoubtedly contributed to the use of “hybrid” as a generic negative description of the seeds associated with the Green Revolution that was noted by Tripp and others. It also forged an enduring connection between hybrid seeds and those that would one day be produced through transgenic manipulation and gene editing.

In other words, it was not a linguistic shift in response to the introduction of commercial F1 hybrids that created terminological confusion about hybrids, as Robert Tripp assumed. Contestation in the 1970s and later over the control of seeds—and with it the shape of global food system—generated the conditions in which “hybrid” would be less a technical description of a seed’s origin and more a shorthand for the style of agricultural production associated with it. For F1 hybrids, Green Revolution varieties, *and* GM crops, many people assumed this meant inputintensive and capital-dependent production that placed industry interests first.

This is an ironic outcome, to say the least. The foundation of arguments like Kloppenburg’s, which equated the control enabled by of F1 hybrid production with that afforded by plant variety protection or patents, depended on an appreciation of the biological and technical specifics of F1 hybrid seed production. But the shorthand that runs roughshod over these specifics clearly endures—and is unlikely to be upended by more rigorous education in the breeding methods that produced them.

## Footnotes

1 See, e.g., Ted Steinburg, *Down to Earth: Nature’s Role in American History* (Oxford: Oxford University Press, 2002); Mark B. Tauger, *Agriculture in World History* (Oxford: Routledge, 2011); Christopher Isett and Stephen Miller, *The Social History of Agriculture: From the Origins to Current Crisis* (Lanham, MD: Rowman & Littlefield, 2017); Alan I. Marcus and Howard P. Segal, *Technology in America: A Brief History*, 3^rd^ edition (London: Palgrave, 2018).

2 Jack Kloppenburg, Jr, *First the Seed: The Political Economy of Plant Biotechnology, 1492–2000*, 2nd edition (Madison: University of Wisconsin Press, 2004); Deborah Fitzgerald, *The Business of Breeding: Hybrid Corn in Illinois, 1890–1940* (Ithaca: Cornell University Press, 1990); Deborah Fitzgerald, “Farmers Deskilled: Hybrid Corn and Farmers’ Work,” *Technology and Culture* 34, no. 2 (1993): 324–343; Philip H. Howard, “Visualizing Consolidation in the Global Seed Industry: 1996–2008,” *Sustainability* 1, no. 4 (2009): 1266–1287.

3 The description “high yielding varieties,” another catch-all for these crop varieties, predominates in many discussions today. For examples of their being described as “hybrids” over several decades, see A. K. Chakravarti, “Green Revolution in India,” *Annals of the Association of American Geographers* 63, no. 3 (1973): 319–330, on p. 319 and 320; Jimoh Omo-Fadaka, “Development: The Third Way.” *Alternatives* 1, no. 1 (1975): 23–53, pp. 29 and 30; Vandana Shiva, *The Violence of the Green Revolution: Third World Agriculture, Ecology, and Politics*, 2nd Impression (London: Zed Books, 1993), on pp. 62 and 119; Carol B. Thompson, “Africa: Green Revolution or Rainbow Evolution?” *Review of African Political Economy* 34, no. 113 (2007): 562–565, on p. 563.

4 E.g., Susan George, *Feeding the Few: Corporate Control of Food* (Washington, DC: IPS, 1979); Cary Fowler and Pat Mooney, *Shattering: Food, Politics, and the Loss of Genetic Diversity* (Tucson: University of Arizona Press, 1990); Vandana Shiva and Kunwar Jalees, *Seeds of Suicide: The Ecological and Human Costs of Seed Monopolies and Globalisation of Agriculture*, revised fourth edition (New Delhi: Navdanya, 2006). Further examples are discussed below.

5 Robert Tripp, “Biodiversity and Modern Crop Varieties: Sharpening the Debate,” *Agriculture and Human Values* 13, no. 4 (1996): 48–63.

6 Ibid, p. 51. See also Kloppenburg (op cit.), p. 68.

7 Clare Coleman, “Plant Hybridity Before Mendelism: Diversity and Debate in British Botany, 1837 – 1899,” PhD thesis, University of Leeds, 2021. See more extended discussions of hybrids as an object of interest among botanists and breeders across history in Noel Kingsbury, *Hybrid: The History and Science of Plant Breeding* (Chicago: University of Chicago Press, 2011).

8 I do not attempt to link this with the literature on hybridity as it relates to human social, cultural, and genetic mixing, e.g. Avtar Brah and Annie E. Coombes, eds., *Hybridity and its Discontents* (London: Routledge, 2000); John Hutnyk, “Hybridity, Ethnic and Racial Studies,” 28, no. 1 (2005): 79–102; Peter Burke, *Cultural Hybridity* (Cambridge: Polity, 2009). For reflections on maize research in relation to human diversity, see Helen Anne Curry, “Taxonomy, Race Science, and Mexican Maize,” *Isis* 112, no. 1 (2021): 1–22.

9 The 2021 catalog is available along with many past editions on the Seed Savers Exchange website. Seed Savers Exchange, last accessed 23 August 2021, https://www.seedsavers.org/. An example of an account that relies on this contrast is Janisse Ray, *The Seed Underground: A Growing Revolution to Save Food* (White River Junction, Vermont: Chelsea Green Publishing, 2012).

10 This points to an important role of journalist or media presentations of crop biotechnologies in the trajectory of “hybrid” I trace here although I do not address it in this essay. Some typical examples of these explanatory webpages include E. Vinje, “Hybrids, GMOs: Not the Same,” Planet Natural Research Center, last accessed 27 August 2021, https://www.planetnatural.com/hybrid-gmo/; Vicki Mattern, “Hybrid Seeds vs. GMOs,” Mother Earth News, last accessed 27 August 2021, https://www.motherearthnews.com/real-food/hybrid-seeds-vs-gmos-zb0z1301zsor; and Richard Brogren, “Hybrids and GMO Seeds,” LSU AgCenter, last accessed 27 August 2021, https://www.lsuagcenter.com/profiles/jmorgan/articles/page1536932413568.

11 See, e.g., A. Richard Crabb, *The Hybrid-Corn Makers: Prophets of Plenty* (New Brunswick, NJ: Rutgers University Press, 1947).

12 Donald Duvick, “Biotechnology in the 1930s: The Development of Hybrid Maize,” *Nature Reviews Genetics* 2 (2001): 69–74, on p. 71.

13 Donald Duvick, “Heterosis: Feeding People and Protecting Natural Resources,” in *The Genetics and Exploitation of Heterosis in Crops*, edited by James G. Coors and Shivaji Pandey (Madison, WI: American Society of Agronomy, 1999), 19–29, on p. 20.

14 James F. Crow, “90 Years Ago: The Beginning of Hybrid Maize,” *Genetics* 148 (1998): 923–928, on p. 923.

15 The varying degrees of success achieved in producing hybrids of different crops by the end of the twentieth century is documented in *Genetics and Exploitation of Heterosis in Crops* (op cit.).

16 Pushpendra Kumar Gupta, Harindra Singh Balyan, Vijay Gahlaut, Gautam Saripalli, Bijendra Pal, Bhoja Raj Basnet, and Arun Kumar Joshi, “Hybrid Wheat: Past, Present, and Future,” *Theoretical and Applied Genetics* 132 (2019): 2463–2483.

17 Jiming Li and Longping Yuan, “Hybrid Rice: Genetics, Breeding, and Seed Production,” in *Plant Breeding Reviews* 17, edited by Jules Janick (New York: John Wiley and Sons, 2000), pp. 15–158.

18 The research organizations associated with the Green Revolution did produce F1 hybrid maize in some cases. However terminological confusion reigns here, too. For example, in Mexico the use of “hybrid” to describe a subset of maize plants that were not F1 hybrids has muddled subsequent historical analyses. See Karin Matchett, “Untold Innovation: Scientific Practice and Corn Improvement in Mexico, 1935–1965,” PhD thesis, University of Minnesota, 2003.

19 See a technical account of these hybridizations in Thomas A. Lumpkin, “How a Gene from Japan Revolutionized the World of Wheat,” in *Advances in Wheat Genetics: From Genome to Field*, edited by Yasunari Ogihara, Shigeo Takumi, and Hirokazu Handa (Tokyo: SpingerOpen, 2015), pp. 13–20.

20 Tripp, “Biodiversity and Modern Crop Varieties,” (op cit.) p. 52.

21 There are good reasons to see these endeavors as historically aligned; see Jonathan Harwood, “Was the Green Revolution Intended to Maximise Food Production?” *International Journal of Agricultural Sustainability* 17, no. 4 (2019): 312–325.

22 Useful entry points into this history include Glenn E. Bugos and Daniel J. Kevles, “Plants as Intellectual Property: American Practice, Law and Policy in World Context,” *Osiris* 7 (1992): 74–104; Cary Fowler, *Unnatural Selection: Technology, Politics and Plant Evolution* (Yverdon, Switzerland: Gordon and Breach, 1994); and Keith Aoki, *Seed Wars: Controversies and Cases on Plant Genetic Resources and Intellectual Property* (Durham: Carolina University Press, 2008).

23 This was linked to broader critiques of capitalism and its relationship to scientific and technological development. For example, industrial food and agriculture were frequently targeted by the Marxist activists associated with the organization Science for the People. See Sigrid Schmalzer, Daniel S. Chard, and Alyssa Botelho, *Science for the People: Documents from America’s Movement of Radical Scientists* (Amherst: University of Massachusetts Press, 2018), ch 6.

24 Rachel Schurman and William A. Munro, “Ideas, Thinkers, and Social Networks: The Process of Grievance Construction in the Anti-Genetic Engineering Movement,” *Theory and Society* 35, no. 1 (2006): 1–38; Rachel Schurman and William A. Munro, *Fighting for the Future of Food: Activists versus Agribusiness in the Struggle over Biotechnology*. Minneapolis: University of Minnesota Press, 2010.

25 Pat Mooney, *Seeds of the Earth: A Private or Public Resource?* (Ottawa: International Coalition for Development Action, [1979]).

26 Mooney, *Seeds of the Earth*, p. 84.

27 Jorge Fernandez-Cornejo et al., “The Seed Industry in U.S. Agriculture: An Exploration of Data and Information on Crop Seed Markets, Regulation, Industry Structure, and Research and Development,” Agriculture Information Bulletin Number 786, USDA ERS, January 2004.

28 Kloppenburg, *First the Seed* (op cit.), see summary statements on p. 128. For a similar analysis see Jean-Pierre Berlan and Richard C. Lewontin, “The Political Economy of Hybrid Corn,” *Monthly Review* 38, no. 3 (1986): 35–47.

29 Bryan L. McDonald, *Food Power: The Rise and Fall of the Postwar American Food System* (Oxford: Oxford University Press, 2017).

30 A. J. Ullstrup, “The Impacts of the Southern Corn Leaf Blight Epidemics of 1970–1971,” *Annual Review of Phytopathology* 10 (1972): 37–50.

31 Mooney, *Seeds of the Earth* (op cit.), pp. 11, 70.

32 Kloppenburg, *First the Seed* (op cit.), p. 122.

33 Kloppenburg, *First the Seed* (op cit.), pp. 279, 282–283.

